# Contribution of central sleep apnea to severe sleep apnea hypopnea syndrome

**DOI:** 10.1007/s11325-023-02776-6

**Published:** 2023-02-28

**Authors:** Guoxin Zhang, Xiaoyun Zhao, Fang Zhao, Jin Tan, Qiang Zhang

**Affiliations:** 1 Department of Geriatrics, Tianjin Medical University General Hospital, Tianjin Geriatrics Institute, Tianjin, China; 2 Chest Clinical College, Tianjin Medical University, Tianjin, 300222 China; 3 Respiratory and Critical Care Medicine Department and Sleep Center, Tianjin Chest Hospital, Tianjin, 300222 China; 4 Tianjin Medical University, Tianjin, China

**Keywords:** Central sleep apnea (CSA), Mixed sleep apnea (MSA), Arrhythmia, Heart failure

## Abstract

**Purpose:**

Central sleep apnea (CSA) is usually distinguished from obstructive sleep apnea (OSA). In fact, CSA is often a component of severe sleep apnea hypopnea syndrome (SAHS), rather than occurring alone. We investigated the clinical characteristics and polysomnography (PSG) parameters of CSA components in patients with severe SAHS.

**Methods:**

The clinical characteristics and PSG parameters were retrospectively analyzed.

**Results:**

Pure or dominant CSA was rare (5% of all patients). Of all patients with CSA, 72% also exhibited other apnea subtypes that contributed to severe SAHS. Among patients with severe SAHS, those with CSA were more likely than others to be older; thinner; exhibit higher prevalences of comorbid coronary heart disease, arrhythmia, and heart failure; a higher apnea/hypopnea index (AHI); mixed apnea index (MAI); an elevated oxygen desaturation index (ODI); and more nighttime oxygen saturation levels < 90%. Multivariate logistic regression analysis revealed that older age, comorbid arrhythmia or heart failure, and an elevated ODI were independently associated with CSA.

**Conclusion:**

Patients who complain of snoring or apnea may be better evaluated by comprehensive PSG prior to treatment if they are old, show greater hypoxia, or suffer from arrhythmia and/or heart failure, because such patients are more likely than others to exhibit CSA.

## Introduction

Obstructive sleep apnea (OSA) is very common and is characterized by recurrent episodes of upper airway collapse leading to complete or partial cessation of airflow even with respiratory effort [1]. By contrast, central sleep apnea (CSA), characterized by respiratory apnea accompanied by a lack of respiratory effort during sleep, is much less common, except in patients with cardiac diseases or those who take opiates [2–4]. Previous studies have focused on patients with dominant CSA, regardless of the severity of sleep apnea hypopnea syndrome (SAHS) [5–7]. However, it is noteworthy that CSA is typically comorbid with OSA, mixed sleep apnea (MSA), and hypopnea. These types of SAHS may constitute a severe phenotype (the apnea/hypopnea index [AHI] is ≥ 30) together, which may be more harmful and deserving treatment. Continuous positive airway pressure ventilation (CPAP) may be used to treat suspected OSA directly. But for those patients with a component of CSA, there may be a greater possibility of complex sleep apnea syndrome or lower adherence to CPAP therapy [8–10]. The home sleep apnea test (HSAT) detects OSA in a cost-effective manner but does not distinguish CSA [8, 11]. To allocate medical resources optimally and to ensure appropriate treatment, it is important to be alert for CSA in patients with SAHS irrespective of which apnea subtype predominates. We therefore explored the clinical characteristics and PSG parameters of patients with severe SAHS with and without CSA regardless of whether or not CSA predominated. We also sought indicators of CSA events in an effort to facilitate appropriate diagnosis and treatment.

## Methods

### Subjects

Patients who complained of snoring or apnea and who underwent overnight PSG in the Sleep Center of Tianjin Chest Hospital were retrospectively enrolled from January 2015 to March 2022. The exclusion criteria were total sleep time < 120 min, long-term sedative or sleeping tablet usage, any prior SAHS diagnosis, and noninvasive positive pressure ventilation treatment. We defined pure CSA as a central apnea index (CAI) ≥ 5 with a respiratory event index of any/all other subtype(s) < 5; dominant CSA as an AHI ≥ 5 and a CAI-to-AHI ratio > 50%; and secondary CSA as an AHI ≥ 5 and a CAI ≥ 5 and a CAI-to-AHI ratio ≤ 50%. When exploring the contributions of CAI to severe SAHS, we divided patients into a wCSA group (AHI ≥ 30 and CAI ≥ 5) and an nCSA group (AHI ≥ 30 and CAI < 5). We recorded sex, age, height, weight, neck and waist circumference, and comorbidities. The body mass index (BMI) was the weight in kilograms divided by the square of the height in meters. Atrial arrhythmia was defined as persistent atrial fibrillation (AF) or atrial flutter. Heart failure was defined by a previous diagnosis via echocardiography, assay of the brain natriuretic peptide (BNP) level, or obvious symptoms.

#### PSG

Overnight PSG yielded electroencephalography data (F_4_/M_1_, C_4_/M_1_, O_2_/M_1_, F_3/_M_2_, C_3_/M_2_, O_1_/M_2_) and also included electrooculography, submental electromyography, bilateral anterior tibialis electromyography, and electrocardiography. Respiratory inductance plethysmography was used to monitor the respiratory effort of thoracoabdominal movement. Airflow was assessed using oronasal thermal and pressure sensors. A finger pulse oximeter was employed to record oxygen saturation. Sleep stages were manually assessed using the 2012 American Academy Sleep Medicine criteria [4]. Apnea was defined as an airflow reduction > 90% of the pre-event baseline for ≥ 10 s; hypopnea was defined as a reduction in airflow ≥ 30% of the pre-event baseline for ≥ 10 s with ≥ 3% oxygen desaturation. OSA and CSA events were defined as apneas in the presence or absence of respiratory effort, respectively. Mild, moderate, and severe SAHS were defined as 5 ≤ AHI < 15 events/h, 15 ≤ AHI < 30 events/h, and AHI ≥ 30 events/h, respectively. The oxygen desaturation index (ODI) was the number of times in which oxygen saturation decreased by 3%/h.

### Statistical analysis

Normally distributed quantitative data are presented as means ± standard deviations and non-normally distributed data are given as medians with interquartile ranges. Qualitative data are expressed as frequencies with percentages. Numerical variables were compared using the independent paired *t*-test and the Mann-Whitney *U*-test for normally and non-normally distributed data, respectively. Categorical variables were compared employing the chi-square or Fisher exact test (as appropriate). Only variables with *P-*values < 0.1 were included in multivariate logistic regression, except cerebrovascular disease. In view of the difference in the prevalence of cerebrovascular disease between the two groups and the important influence of cerebrovascular disease on CSA, cerebrovascular disease (*P =* 0.324) was also included in the multivariate regression analysis. For all analyses, a two-tailed *P* < 0.05 was considered significant. All analyses were performed using SPSS ver. 25 software.

## Results

### Subtypes of patients who underwent PSG

**Fig. 1:**
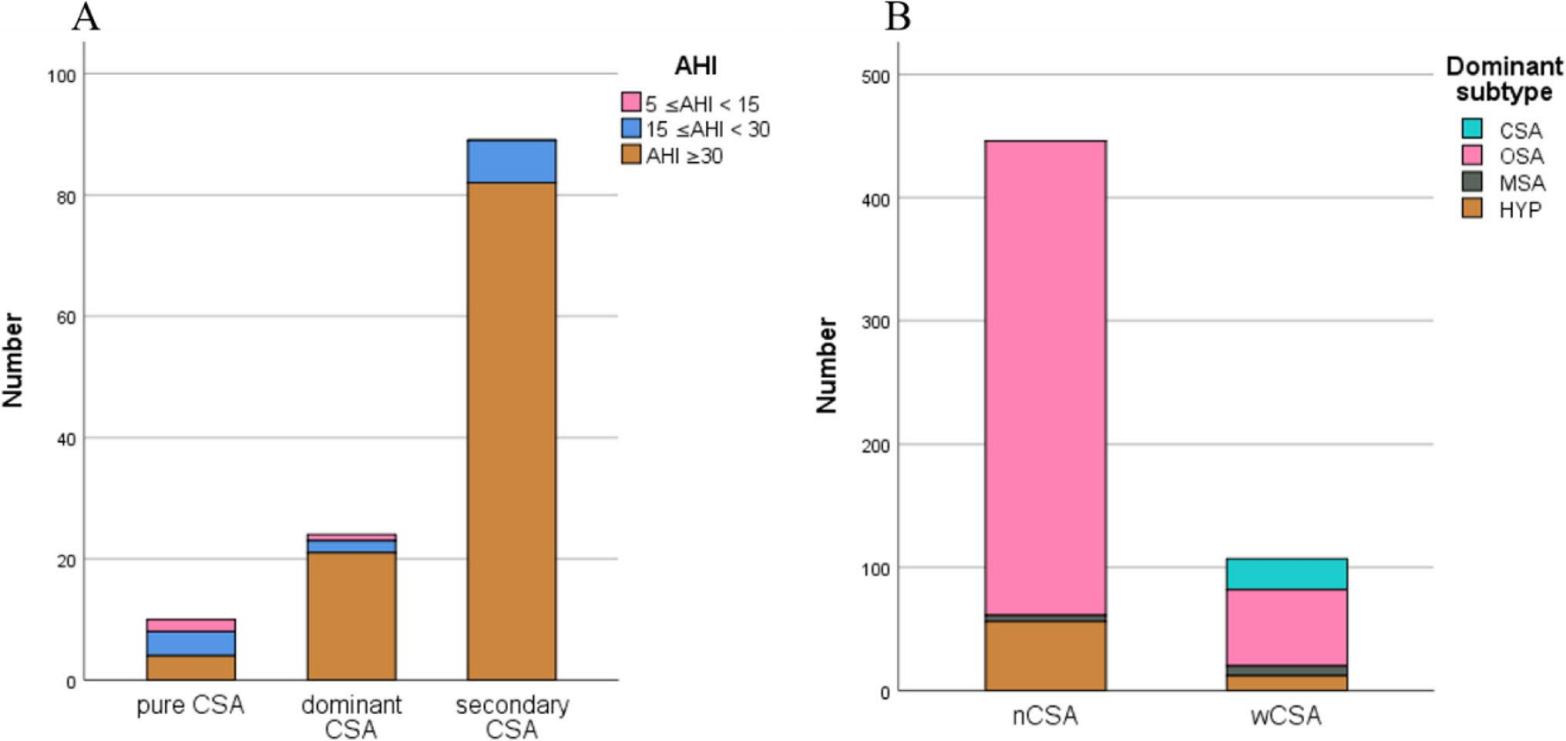
Apnea subtypes of SAHS patients, and SAHS severities. **A** Secondary CSA patients were much more common than pure and dominant CSA patients. The severe SAHS phenotype predominated in both the dominant and secondary CSA groups. **B** 553 patients with severe SAHS (AHI≥30, dominant subtype unspecified) were divided into wCSA group and nCSA group according to CSA existed (CAI≥5) or not. Patients lacking CSA (nCSA) were much more common than those with CSA (wCSA). The OSA subtype predominated in both groups, followed by hypopnea (HYP), and then MSA, grouping variable CSA not included

Of 714 patients who met the inclusion criteria, 123 (17%) had CSA and 3 (2%), 13 (11%), and 107 (87%) for mild, moderate, and severe SAHS, respectively. In all, 10 patients (8%) had pure CSA, 24 (20%) had dominant CSA, and 89 (72%) had secondary CSA (Figure 1A). Thus, most CSA events were comorbid with other apnea subtypes; such combinations constituted severe SAHS. In 553 patients with severe SAHS, 107 (19%) exhibited CSA (CAI ≥ 5, defined as wCSA group) but 446 (80%) did not (CAI < 5, defined as nCSA group). In the wCSA group with severe SAHS, dominant CSA accounted for only 20% of patients (21/107). Among patients with severe SAHS, both with and without CSA, obstructive apneas were the most common type of events (Figure 1B).

### Characteristics, comorbidities, and PSG parameters

We included 553 patients with severe SAHS. Most were males (466, 84%) of mean age 52.7 ± 12.1 years with a median AHI of 51.6 (39.2, 64.4)/h. Patients with (compared to without) CSA were older, thinner, and more likely to exhibit comorbid coronary heart disease, arrhythmia, and heart failure (all *P* < 0.05). Sex and neck and waist circumference did not differ between the two groups; neither did the prevalence of diabetes, cerebrovascular or chronic airway inflammatory disease, or pulmonary embolism (Table 1). Compared to the nCSA group, wCSA patients had a higher AHI and MAI but a lower OAI and hypopnea index (HI). The REM and NREM percentages of total sleep time (TST) did not significantly differ. The percent of night oxygen saturation < 90% (Tsat90) was somewhat greater in the wCSA group, but the ODI was significantly higher (Table 2).

**Table 1:** Characteristics and comorbidities of the nCSA and wCSA groups

Parameter	wCSA (*n* = 107)	nCSA (*n* = 446)	*P-*value
Demographic characteristics			
Males, N (%)	91 (85)	375 (84)	0.805
Age, M ± SD (years)	56.2 ± 11.5	51.8 ± 12.1	0.001^**^
Anatomical characteristics, m (P25, P75)			
BMI	28.8 (26.2, 31.5)	29.7 (27.2, 32.9)	0.036^*^
Neck circumference	42.0 (40.0, 45.0)	43.0 (40.0, 45.0)	0.465
Waist circumference	105.0 (98.0,112.0)	106.0 (99.0, 114.0)	0.500
Comorbidities, N (%)			
Hypertension	55 (51)	185 (42)	0.063
Coronary heart disease	34 (32)	95 (21)	0.021^*^
Arrhythmia	22 (21)	35.(8)	<0.001^***^
Heart failure	5 (5)	2 (0)	<0.001^***^
Diabetes	14 (130)	57 (13)	0.933
Cerebrovascular disease	1 (1)	14 (3)	0.324
Chronic airway inflammatory disease	2 (2)	22 (5)	0.196
Pulmonary embolism	3 (3)	7 (2)	0.416

M ± SD = mean ± standard deviation, m = median, P25 = 25th percentile, P75 = 75th percentile. ^*^P < 0.05, ^**^P < 0.01, ^***^P < 0.001 vs. the nCSA group

**Table 2:** PSG parameters of the nCSA and wCSA groups

PSG parameter	wCSA (*n* = 107)	nCSA (*n* = 446)	*P-*value
AHI	55.1 (42.5, 65.3)	50.7 (38.2, 64.2)	0.039^*^
CAI	10.2 (6.9, 21.1)	0.2 (0, 1.4)	<0.001^***^
REM-CAI	11.1 (3.1, 28.3)	0 (0, 1.1)	<0.001^***^
NREM-CAI	10.4 (6.6, 20.7)	0.2 (0, 1.3)	<0.001^***^
OAI	22.5 (10.4, 38.1)	37.6 (24.6, 53.0)	<0.001^***^
REM-OAI	14.4 (6.5, 29.7)	37.0 (22.2, 51.6)	<0.001^***^
NREM-OAI	18.9 (8.8, 34.4)	38.2 (22.8, 50.2)	<0.001^***^
MAI	5.5 (2.0, 14.4)	0.7 (0.1, 2.7)	<0.001^***^
REM-MAI	2.2 (0.0, 11.7)	0.0 (0.0, 2.7)	<0.001^***^
NREM-MAI	3.8 (1.3, 13.9)	0.3 (0.0, 2.9)	<0.001^***^
HI	4.9 (2.1, 10.8)	7.3 (3.2, 14.1)	0.015*
REM-HI	3.5 (0.5, 11.8)	6.0 (1.1, 14.5)	0.133
NREM-HI	5.9 (2.2, 12.9)	7.4 (3.0, 15.5)	0.183
Percent REM	18.2 (6.3, 26.1)	14.2 (4.8, 22.6)	0.052
Percent NREM	81.8 (73.9, 93.7)	85.7 (77.4, 95.2)	0.050
Mean SpO_2_(%)	92.6 (90.8, 94.0)	93.0 (91.6, 95.0)	0.120
REM- Mean SpO_2_(%)	92.0 (89.9, 93.9)	92.9 (90.6, 94.5)	0.025^*^
NREM-Mean SpO_2_(%)	92.5 (91.0,94.0)	93.2 (91.6, 95.0)	0.094
Lowest SpO_2_(%)	75.0 (66.0, 81.0)	76.0 (66.0, 81.0)	0.945
Tsat90 (%)	16.7 (3.9, 31.4)	9.5 (3.0, 26.4)	0.043^*^
Tsat80 (%)	0.2 (0, 2.9)	0.2 (0, 2.5)	0.994
ODI	43.0 (31.7,57.8)	37.0 (23.3, 54.4)	0.007^**^

Data are shown as medians (P25, P75). *AHI*, apnea/hypopnea index; *CAI*, central apnea index; *OAI*, obstructive apnea index; *MAI*, mixed apnea index; *HI*, hypopnea index; *REM*, rapid eye movement; *NREM*, non-rapid eye movement; *SpO*_*2*_, pulse oxygen saturation; *Tsat90*, percentage of cumulative night time at an oxygen saturation less than 90%; *ODI*, oxygen desaturation index. ^*^P < 0.05, ^**^P < 0.01, ^***^P < 0.001 vs. the nCSA group

### Correlations between the CSA and other parameters

**Fig. 2:**
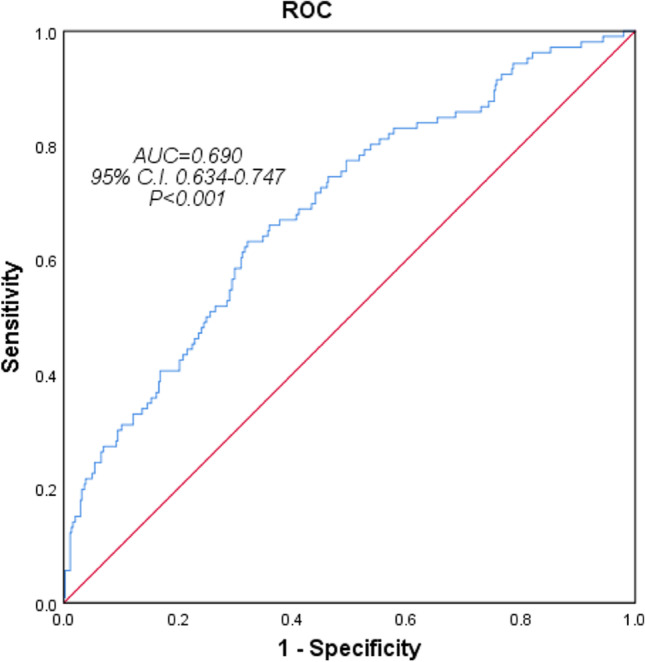
ROC for the four variables combined

Only variables with *P*-values < 0.1 in univariate analysis were included in multivariate logistic regression analysis, with the exceptions of the MAI and HI (only the AHI was included because the AHI is strongly associated with the MAI, CAI, and HI), and exceptions of cerebrovascular disease due to the reasons mentioned above. Older age, a greater ODI, arrhythmia, and heart failure independently predicted comorbid CSA in patients with severe SAHS (Table 3). The area under the receiver operator curves for these four variables (combined) was 0.690 (95% confidence interval 0.634–0.747, *P* < 0.001). The predictive sensitivity was 62.3% and the specificity was 68.3% (Figure 2).

**Table 3:** Association of CSA events with clinical and PSG parameters: results of multivariate logistic regression analysis

Variables	OR	95% *C.I.*		*P*
Age	1.04	1.01	1.06	0.001^**^
BMI	1.01	0.99	1.02	0.606
Hypertension	1.17	0.71	1.91	0.537
Coronary heart disease	1.02	0.57	1.82	0.952
Arrhythmia	2.16	1.08	4.33	0.030^*^
Heart failure	12.19	1.88	79.08	0.009^*^
cerebrovascular disease	0.17	0.02	1.51	0.110
ODI	1.02	1.00	1.04	0.022^*^
AHI	1.01	0.99	1.03	0.606
Percent-REM	2.07	0.39	10.98	0.395
Tsat90	0.99	0.97	1.00	0.085

*C.I.*, confidence interval. ^*^P < 0.05, ^**^P < 0.01 vs. the nCSA group

## Discussion

We found that true CSA (defined as pure and dominant CSA in this article) was rare; the prevalence was 5% (34/714) in our general cohort. The majority of patients with CAIs ≥ 5 exhibited complex phenotypes with CSA components; they had severe SAHS. Patients with CSA differed from others in terms of age, BMI, comorbidities (coronary heart disease, arrhythmia, heart failure), the respiratory event indices, and the extent of oxygen desaturation.

A retrospective study in military veterans indicated that the prevalence of CSA was 0.6%, of which 64.1% was comorbid with OSA [5]. Lucas et al. reported a CSA prevalence of 0.9% in a community population [7], but 1.8% for males and 2.7% for males aged ≥ 65 years. Bixler et al*.* studied community males; the CSA prevalence was low (0.4%) but increased monotonically with age [12]. A clinical study reported a primary CSA prevalence of 3.8%, 64% of which was severe [6]. The prevalence has varied across studies. Clinical studies have reported higher prevalences than community-based works. Several studies have found that CSA patients are more likely to be male than those with OSA [5, 12, 13]. In our study, males were much more common than females in both groups, but the proportions were similar in the two groups (85.0 vs. 84.1%, *P* = 0.805), perhaps because wCSA features many OSA components. Several studies have found that CSA patients are thinner than controls [5, 7]. We similarly found that the BMI of the wCSA group was lower than that of the nCSA group (28.76 vs. 29.70 kg/m^2^, *P* = 0.026).

OSA increases the risk for and progression of hypertension, type 2 diabetes, cardiovascular stroke, and atrial fibrillation [1, 14–17]. Strong associations between CSA and arrhythmias and heart failure have recently been reported. Harmon et al. found that the prevalence of OSA in AF and non-AF groups was similar (54.7 vs. 52.0%, *P* = 0.56) [18]. CSA was more common in their AF group (12.3 vs. 4.4%, *P* = 0.002). A prospective study showed that CSA (odds ratio [OR] 2.58, 95% CI 1.18–5.66) and CSA with Cheyne-Stokes breath (CSB) (OR 2.27, 95% CI 1.13–4.56), but not OSA, predicted incident atrial fibrillation [19]. The association was much stronger in patients aged ≥ 76 years. Another study drew similar conclusions: CSA and CSB were significantly associated with AF (OR 5.15, 95% CI 2.21–12.52 and OR 6.26, 95% CI 2.05–19.14, respectively) [20]. However, OSA was not significantly associated with AF. Grimm et al. found that the prevalence of CSA (AHI ≥ 15) was 43% in a cohort with left ventricular ejection fraction (LVEF) ≤ 50%. AF was strongly associated with severe CSA [21]. In a cohort of heart-failure patients with reduced or preserved LVEF, the LVEF correlated negatively with the CAI (*r* = −0.558, *P* < 0.001) but not with the OAI [22]. Thus, CSA is associated more strongly with AF and HF than OSA, despite the high prevalence of OSA in AF and HF patients, perhaps because OSA per se is common in general populations. Our data are consistent with prior data. Compared to patients lacking CSA, the prevalences of arrhythmia (21 vs. 7.8%, *P* < 0.001) and heart failure (5 vs. 0.4%, *P* < 0.001) were higher in those with CSA.

In groups with and without CSA, OSA was the major component, and (unsurprisingly) the OAI was higher in the nCSA group. Notably, the MAI was significantly higher in the wCSA group, indicating a strong association with CSA. Mixed apnea features both central and obstructive components, and is usually considered an obstructive event [23]. Yamauchi et al. concluded that MSA is more closely associated with CSA than OSA, as revealed by instability of respiratory control; the cited authors studied respiratory signals and CPAP acceptance and compliance [24]. Yang et al. [25] found that MSA is linked to both a reduced mean oxygen saturation and the lowest oxygen saturation recorded, similar to what we found. Therefore, we hypothesize that both CSA and MSA are related to respiratory control unstable. We plan to investigate the potential for correlations between CSA and MSA events, and the loop gain and awakening threshold.

Regrettably, we did not categorize hypopnea into central or obstructive hypopnea using the 2012 Criteria of the American Academy of Sleep Medicine. In the clinic, we find it difficult to handle the inspiratory flattening/thoracoabdominal paradox. We may thus have underestimated central events. Central or obstructive events are optimally and precisely detected via esophageal, electrode catheter manometry [26], but patients dislike this.

As is true of all retrospective investigations, some patient data were lacking. In addition, selection bias may be in play; this would overestimate CSA events. However, this is the first work to view CSA events as components of severe SAHS; we did not focus on pure CSA. Patients with and without CA differed in terms of demography, complications, and PSG parameters. Physicians should have raised suspicion when patients are old, exhibit low oxygen saturation during sleep, and are comorbid with arrhythmia or heart failure.

In conclusion, CSA patients (compared to others) were older, more hypoxic, and more likely to suffer from arrhythmia and heart failure. Both MSA and CSA may be related to respiratory control instability. We recommend that patients with the above-mentioned risk factors undergo PSG rather than HSAT for the accurate diagnosis and appropriate therapy.

## Data Availability

The datasets generated during and/or analysed during the current study are available from the corresponding author on reasonable request.
